# Supplementary material for: Ku-Band Mixers Based on Random-Oriented Carbon Nanotube Films
Source: Nanomaterials (Basel). 2024 Feb 29;14(5):450. doi: 10.3390/nano14050450 (PMC10935208; doi:10.3390/nano14050450)
Supplement: Supplementary file 1 [file nanomaterials-14-00450-s001.zip › nanomaterials-2828164-supplementary.pdf]

Supplementary Materials

Ku-Band Mixers Based on Random-Oriented Carbon Nanotube Films

Mengnan Chang[1,†], Jiale Qian[2,†], Zhaohui Li[1,†], Xiaohan Cheng[3], Ying Wang [1,*], Ling Fan[1], Juexian Cao[2] and Li Ding [4,*]

[1] Key Laboratory of Luminescence & Optical Information, Ministry of Education, School of Physical Science and Engineering, Beijing Jiaotong University, Beijing 100044, China;
[2] Hunan Institute of Advanced Sensing and Information Technology, Xiangtan University, Hunan 411105, China;
[3] Academy for Advanced Interdisciplinary Studies, Peking University, Beijing 100871, China;
[4] Key Laboratory for the Physics and Chemistry of Nanodevices and Center for Carbon-Based Electronics, School of Electronics, Peking University, Beijing 100871, China;
† Mengnan Chang, Jiale Qian and Zhaohui Li contributed equally to this work;
* Correspondence: yingw@bjtu.edu.cn, lding@pku.edu.cn.

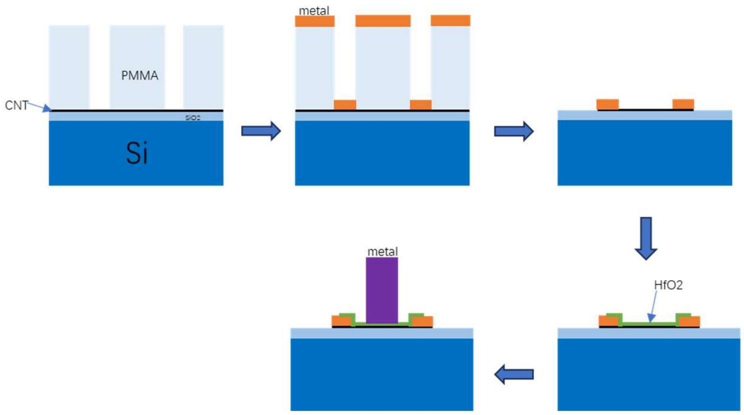

Figure S1. Schematic diagram of device fabrication process.

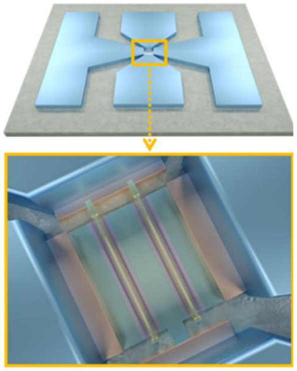

(a) (b)

Figure S2. Structure and DC characteristics of a two-finger configuration RF transistor based on random-oriented carbon nanotube films: (a) Schematic diagram of the structure of a two-finger CNT based RF transistor with a total channel width of 20 μm at high magnification SEM; (b) Typical transfer characteristics curve of a 50 nm gate length two-finger RF transistor.

(a) (b) (c)

Figure S3. Variation of conversion gain of CNT mixer under different measurement bias conditions: (a) Conversion gain (CG) performance of the CNT mixer vary with gate voltage also with an input RF signal frequency of 10 GHz; Off-state region (gate voltage larger than -0.4 V) with low current and transconductance would induce degraded CG; (b) Conversion gain (CG) performance of the CNT mixer scaling rule on local oscillator (LO) input signal power when the input RF signal frequency is 10 GHz with power of -5 dBm. The best conversion gain is close to -11 dB when PLO reaches to almost 7 dBm; (c) The conversion gain (CG) performance of the CNT mixer at different RF signal frequencies with a local oscillator (LO) signal power of 7 dBm and an RF signal power of -5 dBm. When the RF signal is 10 GHz, 12 GHz and 15 GHz respectively, the conversion gain (CG) of the device could be -11.43 dB, -13.11 dB and -17.53 dB respectively.

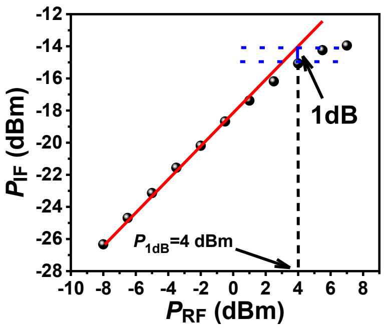

Figure S4. 1 dB compression point measurement. The output power of the CNTs mixer at different RF signal input power levels with a LO (local oscillator) signal power of 5 dBm. The 1 dB compression point (P1dB) of this CNTs mixer is shown to be over 4 dBm.

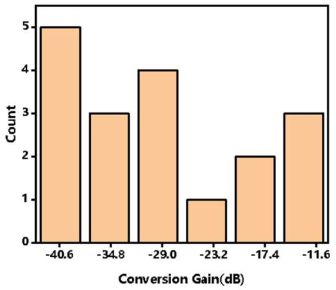

(a)

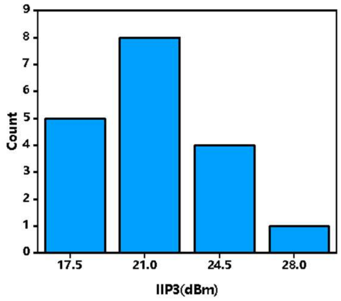

(b)

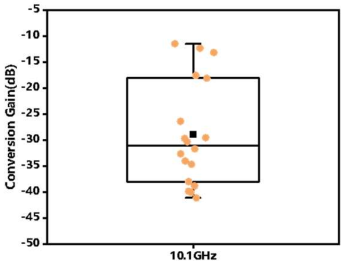

(c)

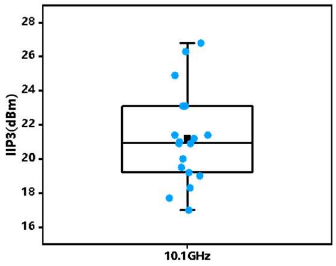

(d)

Figure S5. Statistical results of CNTs-based RF mixers: (a) and (b) are histograms distribution of conversion gain and Input 3rd order intercept point (IIP3); (c) and (d) are box plots of (a) and (b), representing conversion gains of -28.82±10.11 dB and IIP3 of 21.20±2.76 dBm, respectively.

Table S1. Comparison of the performance of CNTs based mixers with other materials mixers.

Frequency (GHz)	CG (dB)	IIP3 (dBm)	Material	Reference
1	-24.5	22.3	CNTs	[1]
1.5	-30.7	N/A	MoS_2	[2]
2	-14	27	Graphene	[3]
2	-12.7	N/A	Graphene	[4]
3.5	-33	21	Graphene	[5]
4	-27	N/A	Graphene	[6]
5	-26	16.5	Graphene	[5]
10	-17	21.5	Graphene	[3]
11	-7.5	10	0.18 μm CMOS	[7]
12	-9.5	17	GaAs	[8]
10	-11.43	18.3	CNTs	This Work
12	-13.11	N/A	CNTs	This Work
15	-17.53	N/A	CNTs	This Work

Extract f_T and f_{max} from S-parameters.

The current gain H_{21} and power gain (Maximum Stable Power Gain, MSG) can be calculated by the following formula:

$$H_{21} = \frac{-2S_{21}}{(1 - S_{11})(1 + S_{22}) + S_{12}S_{21}} \quad (1)$$

$$f_T = f|_{|H_{21}|=1} \quad (2)$$

$$MSG = \frac{|s_{21}|}{|s_{12}|} \quad (3)$$

$$f_{max} = f|_{MSG=1} \quad (4)$$

References:

1. Che, Y.; Lin, Y.-C.; Kim, P.; Zhou, C. T-Gate Aligned Nanotube Radio Frequency Transistors and Circuits with Superior Performance. *ACS Nano* **2013**, *7*, 4343–4350, doi:10.1021/nn400847r.
2. Gao, Q.; Zhang, Z.; Xu, X.; Song, J.; Li, X.; Wu, Y. Scalable High Performance Radio Frequency Electronics Based on Large Domain Bilayer MoS2. *Nat Commun* **2018**, *9*, 4778, doi:10.1038/s41467-018-07135-8.
3. Moon, J.S.; Seo, H.-C.; Antcliffe, M.; Le, D.; McGuire, C.; Schmitz, A.; Nyakiti, L.O.; Gaskill, D.K.; Campbell, P.M.; Lee, K.-M.; et al. Graphene FETs for Zero-Bias Linear Resistive FET Mixers. *IEEE Electron Device Lett.* **2013**, *34*, 465–467, doi:10.1109/LED.2012.2236533.
4. Tian, M.; Li, X.; Gao, Q.; Xiong, X.; Zhang, Z.; Wu, Y. Improvement of Conversion Loss of Resistive Mixers Using Bernal-Stacked Bilayer Graphene. *IEEE Electron Device Lett.* **2019**, *40*, 325–328, doi:10.1109/LED.2018.2889153.
5. Lyu, H.; Wu, H.; Liu, J.; Lu, Q.; Zhang, J.; Wu, X.; Li, J.; Ma, T.; Niu, J.; Ren, W.; et al. Double-Balanced Graphene Integrated Mixer with Outstanding Linearity. *Nano Lett.* **2015**, *15*, 6677–6682, doi:10.1021/acs.nanolett.5b02503.
6. Lin, Y.-M.; Valdes-Garcia, A.; Han, S.-J.; Farmer, D.B.; Meric, I.; Sun, Y.; Wu, Y.; Dimitrakopoulos, C.; Grill, A.; Avouris, P.; et al. Wafer-Scale Graphene Integrated Circuit. *Science* **2011**, *332*, 1294–1297, doi:10.1126/science.1204428.
7. Chen, J.-D.; Lin, Z.-M. 2.4 GHz High IIP3 and Low-Noise Down-Conversion Mixer. In Proceedings of the APCCAS 2006 - 2006 IEEE Asia Pacific Conference on Circuits and Systems; IEEE: Singapore, December 2006; pp. 37–40.
8. HMC220B Datasheet: GaAs, MMIC, Fundamental Mixer, Analog Devices. See the following: https://www.analog.com/media/en/technical-documentation/data-sheets/hmc220b.pdf.
